# A case report on the treatment of autoimmune hemolytic anemia after CBT by daratumumab in a mucopolysaccharidosis type III patient

**DOI:** 10.1097/MD.0000000000044874

**Published:** 2025-09-26

**Authors:** Zhuo-yang Bian, Li Xu

**Affiliations:** a Graduate School, Bengbu Medical University, Bengbu, China; b Department of hematology, Anhui No. 2 Provincial People’s Hospital, Hefei, China.

**Keywords:** autoimmune hemolytic anemia, case report, daratumumab, mucopolysaccharidosis, umbilical cord blood stem cell transplantation

## Abstract

**Rationale:**

Mucopolysaccharidosis type III, also referred to as Sanfilippo disease, is a rare hereditary lysosomal storage illness and one of the more common variants. The illness can affect all tissues and organs of the body, with the central nervous system being the most profoundly impacted. Autoimmune hemolytic anemia (AIHA) is a rare, diverse, antibody-mediated disorder characterized by the production of antibodies that target and destroy red blood cells, leading to their rapid destruction. AIHA is a prevalent and notable complication in recipients following umbilical cord blood stem cell transplantation. Immunosuppressive treatments, including intravenous immunoglobulin and rituximab, have been the primary therapeutic modalities. There is no consensus on the appropriate treatment for post-transplant severe AIHA.

**Patient concerns:**

This article details a 5-year-old male diagnosed with mucopolysaccharidosis type III. He developed AIHA + 144 days after umbilical cord blood stem cell transplantation.

**Diagnoses:**

Genetic testing, cranial CT, blood cell analysis, direct/indirect antihuman globulin test.

**Interventions:**

Treatment encompasses red blood cell transfusion to rectify anemia, intravenous administration of methylprednisolone, calcium gluconate, alkaline solutions, plasma exchange, rituximab, gamma globulin, and more symptomatic therapies. The symptoms of hemolysis were inadequately managed throughout the disease progression and were alleviated upon therapy with daratumumab.

**Outcomes:**

The treatment was conducted without adverse effects, and the problem was well managed. A telephonic follow-up with the patient was conducted by our department 546 days post-discharge. The child’s cognitive ability, linguistic skills, and other neurological processes remained stable without further decline.

**Lessons:**

This report discusses a child with MPS III, a rare disorder characterized by complex and varied phenotypes, including distinctive facial features, short stature, cognitive regression, varying degrees of skeletal deformities, and hepatosplenomegaly. The patient has autoimmune hemolysis after umbilical cord blood transplantation. For patients who do not respond to rituximab and gamma globulin, daratumumab may be considered.

## 1. Introduction

Mucopolysaccharidoses (MPS) type III is an inherited lysosomal storage disorder resulting from the deficiency of various enzymes necessary for the degradation of heparan sulfate, leading to its progressive accumulation in the body and subsequent pathological alterations in the central nervous system and other organs.^[1]^ The inheritance patterns of lysosomal storage diseases and the associated molecular mechanisms contribute to the rarity of different ailments to a certain degree. The majority of mucopolysaccharidoses result from recessive or X-linked inheritance patterns, indicating that the disease manifests only when an individual possesses 2 copies of the pathogenic gene or when a deficiency arises in men.^[2]^ This leads to a reduced prevalence of these illnesses in comparison to prevalent polygenic diseases. The pathogenic processes of lysosomal storage disorders are intricate, involving the disruption of various metabolic pathways. The breakdown of glycosaminoglycans necessitates a sequence of meticulously regulated enzymatic processes, and any mistake at any stage might result in the progressive accumulation of substrates, leading to multisystem clinical manifestations. Nevertheless, this accumulation process frequently advances gradually, and initial symptoms are nonspecific, complicating quick clinical diagnosis for many patients. This indirectly influences the statistics and comprehension of the disease’s prevalence.

The infrequency of this occurrence is attributed to the uncommon nature of the unique genetic mutation, its recessive or X-linked inheritance patterns, and its association with the intricate clinical mechanisms of the disease and delayed diagnosis. daratumumab selectively targets plasmablasts, transient plasma cells, and enduring plasma cells, the latter serving as the primary source of antiplatelet autoantibodies.^[3]^ We successfully treated individuals with MPS type III who developed autoimmune hemolytic anemia (AIHA) after undergoing Umbilical cord blood transplantation (CBT) with daratumumab. The study offers further evidence for the efficacy of daratumumab in managing AIHA patients unresponsive to first or secondary therapies, including Rituximab, while considering their individual conditions.

## 2. Case report

**Figure 1.**
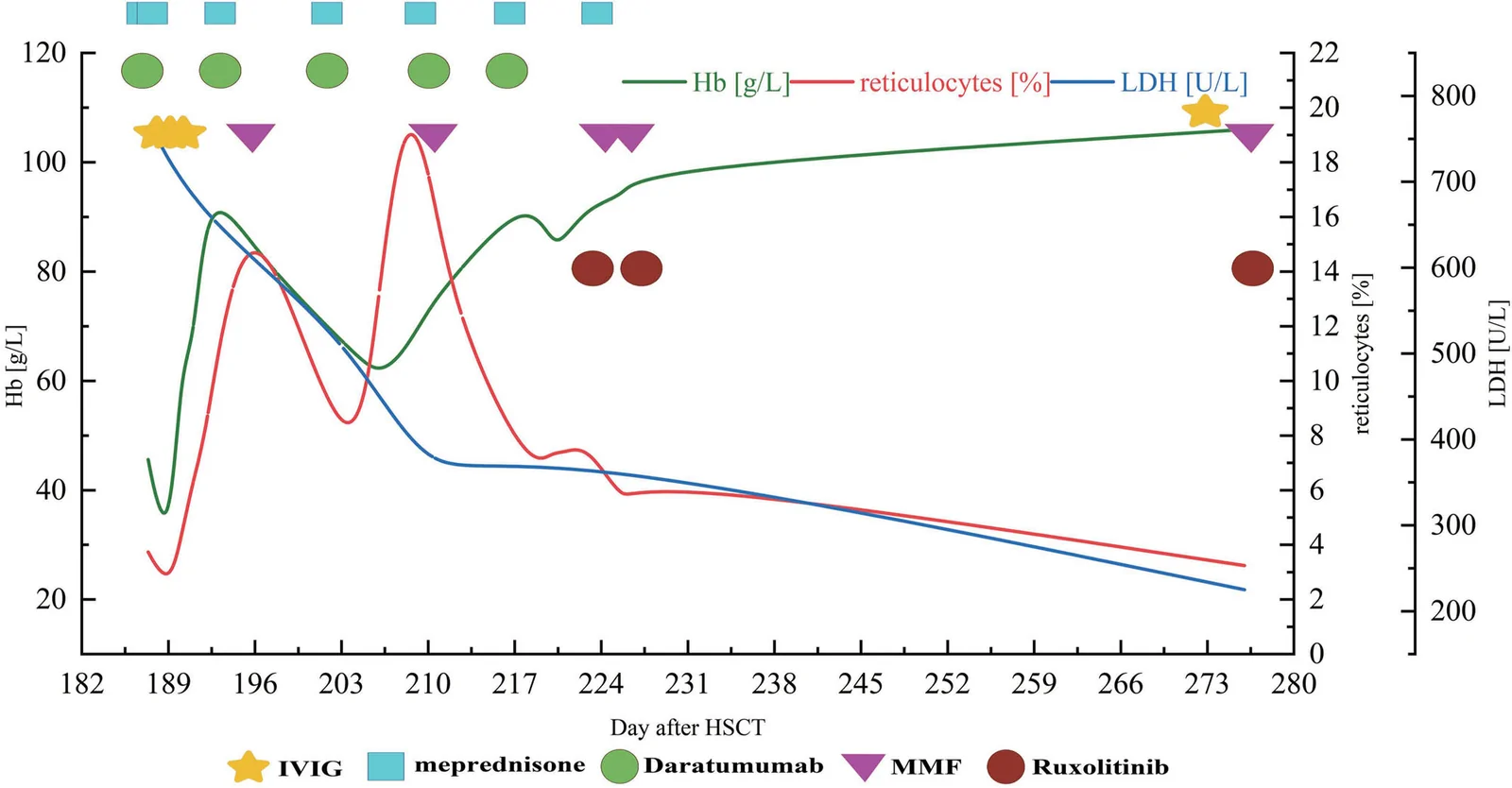
Representation of laboratory parameters and treatment strategies in the patient. IVIG = intravenous immunoglobulin; MMF = mycophenolate mofetil.

The patient is a 5-year-old male diagnosed with l-iduronic aciduria deficiency, who underwent umbilical cord blood stem cell transplantation for MPS type III. He received 3.05 × 10^7^ nucleated cells per kilogram of body weight, maintaining type B blood, Rh(+). Following the administration of prophylaxis with methotrexate and cyclosporine A for graft-versus-host disease, no signs of graft-versus-host disease were observed. With the exception of a little fever, the boy’s post-transplant recovery progressed without difficulties. Neutrophils were fully engrafted by the early morning of day + 27. The patient manifested AIHA 144 days following umbilical blood transfusion. Anemia worsened, exhibiting symptoms of nausea, vomiting, pallor, an abnormal direct antiglobulin test, elevated indirect bilirubin levels, and an increased reticulocyte count. The patient was admitted to the hospital with a body mass of 13.0 kg. He was conscious, oriented, and presented with anemia. There were no signs of jaundice on the skin or mucosa, and superficial lymph nodes were not enlarged. The head was large, and lung auscultation revealed clear breath sounds without dry or wet rales. Cardiac rhythm was synchronous, and no murmurs were detected during valve auscultation. The abdomen was soft, exhibiting no tenderness, rebound pain, or muscle rigidity. Pathological reflexes were absent. Critical values upon admission included hemoglobin at 26 g/L, reticulocyte count at 60.1 × 10^9^/L, lactate dehydrogenase at 876 U/L, indirect bilirubin at 53.3 μmol/L, ferritin at 3630 pmol/L, and a positive direct anti-globulin test. The diagnosis of Coombs-positive AIHA and cytomegalovirus has been confirmed. The patient underwent treatments comprising blood transfusions, plasma exchange, glucocorticoids, immunoglobulins, and rituximab. The decrease in hemoglobin has decelerated; however, levels of indirect bilirubin and LDH continue to be elevated. During the hospitalization, the patient was administered 4 units of irradiated washed red blood cells and a cumulative total of 30 g of immunoglobulin (5 g/vial, delivered via intravenous infusion once daily). Nonetheless, no notable enhancement was detected in the patient’s hemoglobin, bilirubin, or lactate dehydrogenase levels. The hemolytic effects exhibited variability after the first 4 administrations of daratumumab. Methylprednisolone was used in combination with treatment, and red blood cells were transfused if necessary. Following the patient’s fifth injection of daratumumab on day + 211, the child has not needed a blood transfusion for almost 1 week. On day 276 post-umbilical blood reinfusion, during the fourth hospital visit, the patient’s bilirubin, hemoglobin, and lactate dehydrogenase levels were within normal ranges, indicating significant improvement following CD38 treatment, thus confirming its effectiveness. Refer to Figure 1 for further details.

## 3. Review of cases in the literature

To our knowledge, three prior papers presented data on 9 patients. Investigating further instances of mucopolysaccharide storage illnesses through PubMed, utilizing the terms daratumumab in conjunction with HSCT, AIHA, and hemolysis. Identified papers were examined for duplicate patients and cross-references. Four cases were documented in the British Journal of Hematology. The 4 patients had durations of treatment of 44.8, 47, 73.3, and 20.5 months, during which they received rituximab, associated medications, and several transfusions prior to their initial daratumumab treatment at 16, 64, 71, and 4 months, respectively. Following the administration of the initial and final doses of daratumumab to these 4 patients, there was a notable decrease in the number of transfusions (3/1, 1/0, 0/0). These 4 instances exemplify effective and prompt input from AIHA.^[4]^ The American Society of Hematology reported 3 new cases of AIHA following hematopoietic stem cell transplantation in children, in addition to appeals. Reports indicate that the occurrence of this AIHA following stem cell transplantation is typically 2% to 6%.^[5,6]^ It is typically induced by autoimmune hemolysis resulting from immune system problems post-stem cell transplantation, with up to 60% of these patients exhibiting minimal response to first-and second-line treatments, including rituximab.^[7]^ In addition to the patient who succumbed to AIHA recurrence, the other 2 patients underwent 20/0 and 4/1 blood transfusions before and following daratumumab treatment, respectively. Substantial symptoms of hemolysis were observed at the 18 and 13-month follow-ups following daratumumab treatment. Furthermore, Hadassah-Hebrew University Medical Center in Jerusalem, Israel, documented a case of primary myelofibrosis in a newborn attributed to a Vps45 protein shortage. The youngster experienced severe refractory hemolytic anemia and thrombocytopenia following stem cell transplantation. The symptoms were entirely alleviated, and blood cell levels were normalized with daratumumab treatment. The child necessitated an average of 1 to 2 units of red blood cells weekly prior to commencing daratumumab therapy. Following the initial administration of daratumumab at a dosage of 16 mg/kg, hemoglobin and platelet levels increased promptly. This rate is remarkable. Following 6 treatment rounds, the patient’s blood cell count normalized, leading to a gradual cessation of frontline glucocorticoid therapy, with no indications of recurrence for 6 months post-treatment conclusion.^[8]^ The Department of Hematology at the Catholic University of Rome, Italy, reported a case of acute myeloid leukemia in a patient who developed anti-D AIHA 5 months post-HSCT. The treatment commenced with oral prednisone, rituximab, and therapeutic plasma exchange to expedite the reduction of antibody titers. The patient required transfusions due to symptomatic anemia and ongoing hemolytic indicators. The patient received daratumumab at an initial dosage of 16 mg/kg of body weight for 4 weeks, totaling 8 doses. Following the second injection, the antibody titer diminished markedly, and the steroid dosage was reduced. Two weeks subsequent to the final injection of daratumumab, the patient underwent a concluding red blood cell transfusion. Three months post-treatment, he remained independent of transfusions, and a bone marrow analysis revealed a modest rise in the erythrocyte lineage with no signs of illness return.^[9]^ Refer to Table 1 for additional information.

**Table 1:** The course of treatment of patients in the literature.

Traits of patients									
	Patient 1	Patient 2	Patient 3	Patient 4	Patient 5	Patient 6	Patient 7	Patient 8	Patient 9
Age when AIHA is diagnosed (yr)	28	60	44	55	5 mo	68	19	25 mo	20 mo
Gender	M	F	F	F	M	M	F	M	F
Foundational state	B-ALL, ASCT	CMML, ASCT	None	None	VPS45 deficiency, HSCT	AML, HSCT	Pre–B-ALL	XLT/WAS	DNA-ligase IV deficiency
Earlier treatments	Pdn, Ritux, HSA, Cycl, CSA	Pdn, IVIg, Ritux, HAS	Pdn, IVIg, Ritux, HSA, Sple, Cycl, MMF	Pdn, Ritux	Pdn, Ritux, IVIG, solumedrol, abatacept, bortezomib	Pdn, Ritux, plasma-exchange	MPN, rituximab	PDN, rituximab, plasmapheresis	MPN/PDN, rituximab
Fusion of RBC prior to initial daratumumab administration (n)	16	64	71	4	24	–		20	4
Initial dose of daratumumab	44	4	73	20	16 mg/kg/dose	16 mg/kg/dose	16 mg/kg/dose	16 mg/kg/dose	16 mg/kg/dose
Diagnosis by AIHA for the initial daratumumab	8	7	3	5	9	3	17	9	11
Joint therapy	Prednisone	Prednisone	Eltrombopag	Prednisone	Corticosteroids	Deferoxamine, corticosteroids	Bortezomib, sirolimus, MMF, ibrutinib, Cy, ATG	MMF, plasmapheresis	MMF, sirolimus, eculizumab
Uses of daratumumab (n)	6	3	6	6	6	8	11	4	7
Post daratumumab RBC transfusions following the initial/final daratumumab dosage (n/n)	3/1	1/0	0/0	0/0	0/0	0/1	–	–	–
Duration from the last daratumumab dose to following therapy (d)	163	–	156	58	–	–	–	–	–
Post-final daratumumab follow-up (d)	260	492	461	87	186	90	Died	480	390

ASCT = allogeneic hematopoietic stem cell transplant, B-ALL = acute B-lymphoblastic leukaemia, CMML = chronic myelomonocytic leukaemia, CSA = cyclosporine A, Cycl = cyclophosphamide, HSA = hematopoiesis-stimulating agents, IVIg = intravenous immunoglobulin, MMF = mycophenolate mofetil, Pdn = prednisone, Ritux = rituximab, Sple = splenectomy.

## 4. Conclusions

MPS III often begins around the age of 2 and advances swiftly. The ailment is marked by progressive deterioration of the central nervous system. Cognitive Behavioral Therapy may empower people with MPS III to acquire a lasting capacity for enzyme production. The mortality rate associated with transplantation hinders the progress of umbilical cord blood stem cell transplantation.^[10]^

AIHA is a prevalent and clinically significant complication in recipients of CBT. Page et al previously reported that infants who underwent unrelated CBT and exhibited reduced CD4 and/or CD8 cells post-CBT experienced a higher incidence. All risk variables share a common characteristic: insufficient immune reconstitution or immunological dysregulation. Current treatment protocols include corticosteroids, immunoglobulins, cytotoxic immunosuppressants, splenectomy, and rituximab.^[11]^ Glucocorticoids function as the principal therapeutic approach.^[12]^ Alongside rituximab as a targeted therapy, alternative second-line treatment regimens induce significant immunosuppressive responses.^[13]^ Rituximab explicitly targets B cells to reduce autoantibody production, thus alleviating hemolysis caused by autoimmunity.^[14,15]^ About one-third of patients exhibit resistance to rituximab.^[16]^ B cells are activated to differentiate into plasma cells(CD20-, CD38+)that produce autoantibodies. Rituximab demonstrates ineffectiveness against these plasma cells characterized by CD20− and CD38+ expression.^[17]^ The patient developed AIHA following umbilical CBT. The department employs rituximab and gammaglobulin to treat AIHA patients. This study found that rituximab was ineffective, and the patient faced difficulties tolerating intravenous immunoglobulin. Daratumumab is a humanized monoclonal antibody utilized in therapeutic applications. Daratumumab received initial approval in 2015 for treating multiple myeloma and has shown efficacy in this disease. Daratumumab exhibits additional immunomodulatory effects due to the presence of the CD38 glycoprotein on various immune cells, such as natural killer cells, monocytes, B cells, and T cells. Daratumumab’s mechanism of action in AIHA is believed to involve the depletion of CD38-expressing plasma cells responsible for autoantibody production, consequently decreasing hemolysis. Daratumumab targets plasma cells and may enhance the peripheral regulatory T-cell population, potentially mitigating post-transplant autoimmunity. Daratumumab’s dual action presents a compelling option for patients unresponsive to conventional therapies, including rituximab and corticosteroids.

This study administered daratumumab, demonstrating significant effectiveness. No adverse effects were noted during the treatment, and the condition was effectively managed. At 546 days post-discharge, our department conducted a telephone follow-up with the patient. The child’s intelligence, language skills, and other neurological functions remained stable without further decline, and there was a notable improvement in emotional and mental states. The patient expressed satisfaction with the treatment process for rare diseases, noting a sense of unprecedented care and support throughout the experience. The medical team focuses on the disease, the patient’s psychological state, and quality of life, offering psychological support and rehabilitation training to assist patients in rebuilding confidence and reintegrating into society.

Individuals with MPS type III who had undergone CBT and subsequently developed AIHA were effectively treated with daratumumab. However, the evidential quality of individual case reports with inconsistent details is limited, and treatment failures are likely underreported. The short follow-up period renders assessing long-term responses, infection rates, and potential lasting harm impractical. The efficacy of HSCT in children with MPS type III requires assessment through larger sample sizes and extended follow-up periods.

## Acknowledgments

We thank the patient for consenting to this case report’s publication. We appreciate the Department of Hematology of the Anhui No. 2 Provincial People’s Hospital’s support. I sincerely thank my mentor for the selfless help and support provided throughout the process of writing my thesis, which has been incredibly beneficial to me.

## Author contributions

**Conceptualization:** Zhuo-yang Bian.

**Writing – original draft:** Zhuo-yang Bian.

**Writing – review & editing:** Li Xu.
